# Decrease of Obesity by Allantoin via Imidazoline I_**1**_-Receptor Activation in High Fat Diet-Fed Mice

**DOI:** 10.1155/2013/589309

**Published:** 2013-03-31

**Authors:** Hsien-Hui Chung, Kung Shing Lee, Juei-Tang Cheng

**Affiliations:** ^1^Institute of Basic Medical Sciences, College of Medicine, National Cheng Kung University, Tainan City 70101, Taiwan; ^2^Department of Surgery, Kaohsiung Municipal Hsiao-Kang Hospital and Kaohsiung Medical University, Kaohsiung City 81201, Taiwan; ^3^Institute of Medical Science, College of Health Science, Chang Jung Christian University, Guei-Ren, Tainan City 71101, Taiwan

## Abstract

The activation of the imidazoline I_1_-receptor (I_1_R) is known to regulate appetite. Allantoin, an active ingredient in the yam, has been reported to improve lipid metabolism in high fat diet- (HFD-)fed mice. However, the effect of allantoin on obesity remains unclear. In the present study, we investigated the effects of allantoin on HFD-induced obesity. The chronic administration of allantoin to HFD-fed mice for 8 weeks significantly decreased their body weight, and this effect was reversed by efaroxan at a dose sufficient to block I_1_R. The epididymal white adipose tissue (eWAT) cell size and weight in HFD-fed mice were also decreased by allantoin via the activation of I_1_R. In addition, allantoin significantly decreased the energy intake of HFD-fed mice, and this reduction was associated with a decrease in the NPY levels in the brain. However, no inhibitory effect of allantoin on energy intake was observed in db/db mice. Moreover, allantoin lowered HFD-induced hyperleptinemia, and this activity was abolished by I_1_R blockade with efaroxan. Taken together, these data suggest that allantoin can ameliorate energy intake and eWAT accumulation by activating I_1_R to improve HFD-induced obesity.

## 1. Introduction

Obesity is a major health problem. It is a leading cause of metabolic syndrome, and its prevalence worldwide has increased throughout the 21st century [1–3]. Obesity is associated with an increased risk of many complications, such as cardiovascular disease, type 2 diabetes, and certain types of cancer [4, 5]. Physical exercise, diet restriction, and medication are the major ways to improve obesity [6–8], but their effectiveness remains limited. Some studies have shown that certain herbal agents have antiobesity effects [9, 10]. Thus, the development of an alternative agent for the treatment of obesity is necessary. 

Allantoin is known as an active ingredient in the yam (*Dioscorea* spp.) [11]. The yam (*Dioscorea rhizome*) contains ureides, including allantoin, which are used to prevent inflammation [12, 13]. The yam is a common plant that is widely used in agriculture and in the drug industry. Recently, some herbs from the Dioscoreaceae have been shown to improve the symptoms of metabolic diseases via antihyperlipidemic and antioxidative effects [14, 15]. Allantoin has been shown to activate the imidazoline I_1_-receptor (I_1_R) in animal models and cell lines [16]. In addition, allantoin attenuates hyperlipidemia and improves hepatic steatosis via the activation of I_1_R to regulate farnesoid X receptor (FXR), demonstrating that I_1_R is involved in lipid homeostasis [16]. However, the effects of allantoin on obesity remain obscure. Thus, in the present study, we investigated the effect of allantoin on high-fat diet- (HFD-)induced obesity and the potential mechanism(s) underlying its activity. 

## 2. Materials and Methods

### 2.1. Induction of Obesity in HFD-Fed Mice

Eight-week-old male C57BL/6 mice (20–25 g) obtained from the Animal Center of National Cheng Kung University Medical College were housed in a temperature-controlled room (25 ± 1°C) under a 12:12-h light:dark cycle (light on at 06:00 h). The mice were divided into two groups. One group was fed with a standard laboratory diet (3.04 kcal/g), and the other group was fed with a high-calorie diet containing 5.16 kcal/g (TestDiet, Richmond, IN, USA) for 12 weeks to induce obesity and metabolic disorders. The db/db mice were obtained from Japan SLC, Inc. (Shizuoka, Japan). All animal procedures were performed according to the Guide for the Care and Use of Laboratory Animals of the National Institutes of Health, as well as the guidelines of the Animal Welfare Act. 

### 2.2. Measurement of Body Weight and Energy Intake

In preliminary experiments, allantoin (Sigma-Aldrich, St. Louis, MO, USA) decreased the body weight of mice fed a HFD. Its activity increased gradually and reached a stable plateau at 8 weeks. Thus, HFD-fed mice were treated with allantoin three times a day for 8 weeks, and body weight was measured at baseline (0 week) and at the 2nd, 4th, 6th, and 8th weeks of the experiment. In addition, the daily energy intake was calculated based on the consumption of normal chow (3.04 kcal/g) or HFD (5.16 kcal/g).

### 2.3. Measurement of Energy Intake in db/db Mice

Mice were food-deprived overnight for 12 h (8 pm–8 am). The mice were weighed and then intraperitoneally injected with either vehicle or allantoin prior to the provision of food. Each mouse was maintained isolation in a cage. The energy intake was calculated over 4 h as described previously [17]. 

### 2.4. Measurement of Epididymal White Adipose Tissues

At the end of the experimental period, the mice were sacrificed under 3% isoflurane anesthesia. The epididymal white adipose tissues (eWAT) were isolated and weighed. Then, the eWAT ratio was calculated relative to the body weight of each individual.

### 2.5. Immunosorbent Assay for NPY Levels

The hypothalamus was isolated from sacrificed mice, and the NPY concentration was determined. The obtained samples were homogenized at 4°C in ice-cold homogenization buffer containing 10 mM Tris-HCl (pH 7.4), 20 mM EDTA, 10 mM EGTA, 20 mM *β*-glycerolphosphate, 50 mM NaF, 50 mM sodium pyrophosphate, 1 mM phenylmethylsulfonyl fluoride, 25 *μ*g/mL leupeptin, and 25 *μ*g/mL aprotinin—protease inhibitors in a Teflon/glass homogenizer. The homogenate was centrifuged at 6000 ×g for 20 min at 4°C, and the supernatant was used for NPY quantification. The NPY in each sample was measured using a commercially available mouse enzyme-linked immunosorbent assay (Phoenix Europe GmbH, Karlsruhe, Germany). The absorbance was measured in a SpectraMax 340PC ELISA reader (Molecular Devices Corporation, Union City, CA, USA) at 450 nm.

### 2.6. Determination of Leptin Levels

The mice were fasted for 12 h and anesthetized. Blood samples were collected from the retro-orbital sinuses of each group. The blood samples were then centrifuged at 3,000 rpm for 10 min. Then, the leptin concentration was determined using enzyme-linked immunosorbent assay (ELISA). According to the assay procedure, the determination of leptin in samples was carried out using a commercially available mouse ELISA kit (Assaypro, St. Charles, MO, USA). The absorbance was measured by a SpectraMax 340PC ELISA reader (Molecular Devices Corporation, Union City, CA, USA) at 450 nm.

### 2.7. Histological Analysis

The epididymal white adipose tissues were removed from each group of mice and fixed in 10% formaldehyde at 4°C for 2 days. Fixed specimens were dehydrated and embedded in paraffin. The specimens were then cut into 5 *μ*m thick sections at 50 *μ*m intervals and stained with hematoxylin and eosin (H and E; Muto Pure Chemicals, Tokyo, Japan). The sections were observed under a light microscope.

### 2.8. Statistical Analysis

All values are presented as the mean ± SEM from one group of animals or samples. Analysis of variance and Dunnett's post hoc test were used to evaluate any significant differences between groups. *P* < 0.05 was considered to indicate a significant difference.

## 3. Results

### 3.1. The Effect of Allantoin on Body Weight in HFD-Fed Mice

**Figure 1:**
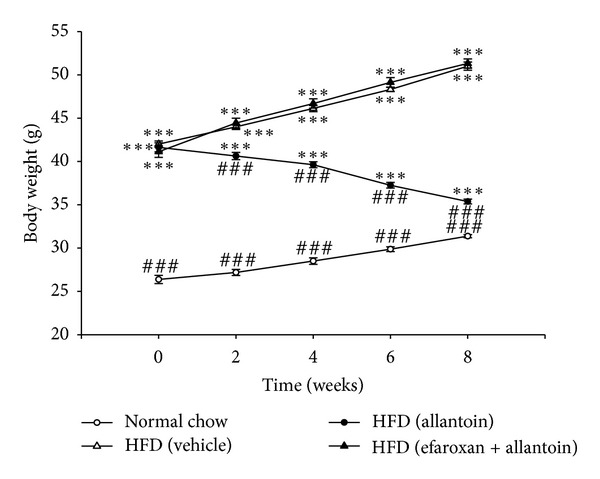
The inhibitory effect of I_1_R antagonist—efaroxan on the allantoin-induced decrease in body weight in HFD-fed mice. Efaroxan (1.5 mg/kg) was intraperitoneally injected 30 min before the injection of allantoin (5 mg/kg). The values are expressed as the mean ± SEM from each group of eight animals ***P < 0.001 compared with the normal chow-fed group at the same time point; ^###^ *P* < 0.001 compared with the HFD-fed group at the same time point.

As shown in Figure 1, mice that were fed with the HFD for 3 months showed a marked (*P* < 0.05) increase in body weight (42.00 ± 0.34 g, *n* = 8) compared with the normal chow-fed mice (26.38 ± 0.44 g, *n* = 8). The average body weight of HFD-fed mice that received intraperitoneal injections of allantoin (5 mg/kg) three times a day for 8 weeks was significantly reduced compared with that of the vehicle-treated HFD-fed mice (35.38 ± 0.26 g versus 51.00 ± 0.44 g, *n* = 8). The allantoin-induced decrease in body weight was attenuated by the intraperitoneal injection of efaroxan at a dose (1.5 mg/kg) sufficient to block imidazoline I_1_-receptors [17].

### 3.2. Improvement of Epididymal White Adipose Tissue (eWAT) in HFD-Fed Mice by Allantoin

**Figure 2:**
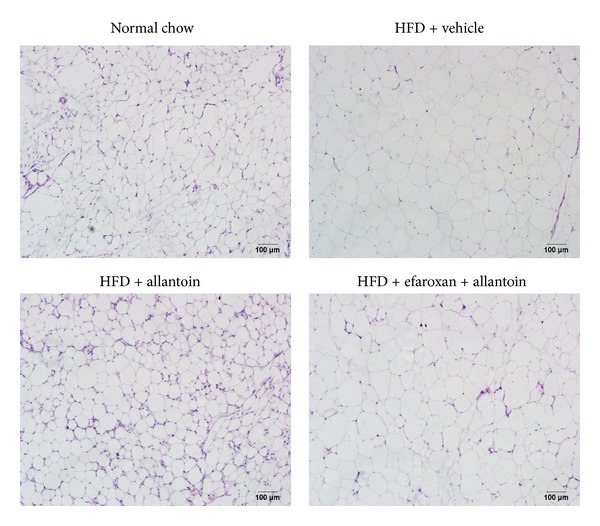
Treatment with allantoin improves epididymal white adipose tissue (eWAT) in high fat diet-fed mice. The mice were fed a high fat diet (HFD) for 12 weeks. Then, allantoin (5 mg/kg) was intraperitoneally injected into the HFD-fed mice three times daily for 8 weeks. In another group, 1.5 mg/kg efaroxan was also injected into mice at 30 min before the injection of allantoin. At the end of the experiment, eWAT was isolated from each mouse. Histological evaluation of the eWAT was performed by staining with hematoxylin-eosin (magnification: ×200).

As shown in Figure 2, the HFD significantly induced obesity in mice. The epididymal white adipose tissue (eWAT) cell size in HFD-fed mice was larger than that in normal chow-fed mice. Allantoin ameliorated these changes in eWAT. Pretreatment with efaroxan reversed the distribution and types of eWAT to that of the HFD group, indicating that the activity of allantoin was abolished by efaroxan (Figure 2). Allantoin also decreased the eWAT weight, and this reduction could be reversed by pretreatment with efaroxan. The eWAT ratio also exhibited the same pattern (Table 1).

### 3.3. The Involvement of Imidazoline I_1_-Receptors in the Allantoin-Induced Reduction of Energy Intake

**Figure 3:**
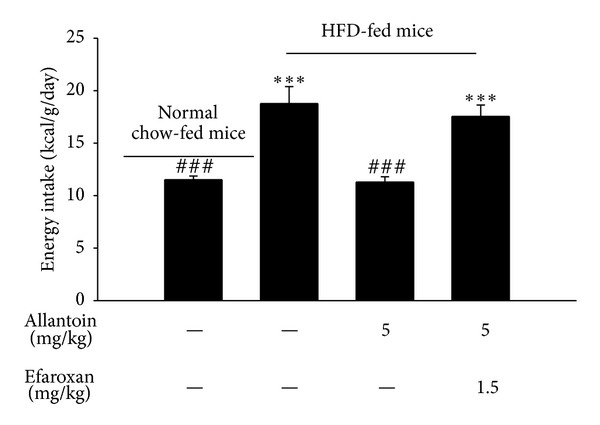
The inhibitory effect of allantoin on energy intake in HFD-fed mice. Efaroxan (1.5 mg/kg) was administered 30 min before the intraperitoneal injection of allantoin (5 mg/kg). The value showed the mean ± SEM of eight animals. ***P < 0.001 compared with the normal chow-fed group; ^###^ *P* < 0.001 compared with the vehicle-treated HFD-fed group.

Intraperitoneal injection of allantoin (5 mg/kg) three times a day for 8 weeks markedly reduced the energy intake of HFD-fed mice, from 18.77 ± 1.52 kcal/g/day to 11.29 ± 0.47 kcal/g/day (Figure 3). Pretreatment with efaroxan (1.5 mg/kg, i.p.) abolished this activity. However, treatment with efaroxan alone had no influence on the energy intake of HFD-fed mice.

### 3.4. Changes in the Neuropeptide Y (NPY) Level in the Hypothalamus of HFD-Fed Mice

**Figure 4:**
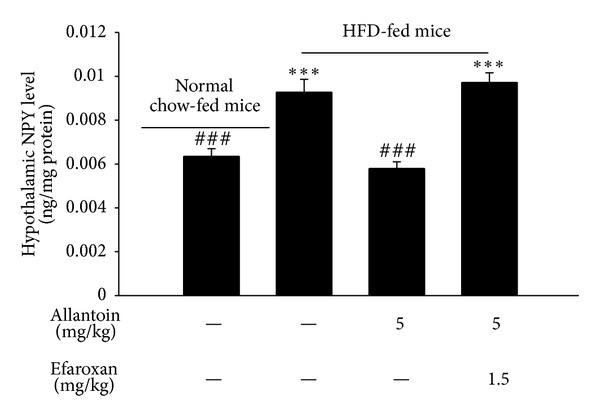
Changes in the hypothalamic NPY level in HFD-fed mice treated with allantoin for 8 weeks. HFD-fed mice received continuous administration of allantoin (5 mg/kg, i.p. three times per day), while another group was pretreated with efaroxan (1.5 mg/kg) 30 min before the administration of allantoin. The values are expressed as the mean ± SEM from each group of eight animals ***P < 0.001 compared with the normal chow-fed group; ^###^ *P* < 0.001 compared with the vehicle-treated HFD-fed group.

As shown in Figure 4, the hypothalamic NPY level in HFD-fed mice was markedly reduced by treatment with allantoin (5 mg/kg, i.p.) for 8 weeks. Pretreatment with efaroxan (1.5 mg/kg i.p.) 30 min before the administration of allantoin abolished this change in hypothalamic NPY (Figure 4).

### 3.5. Allantoin Activity in db/db Mice

**Figure 5:**
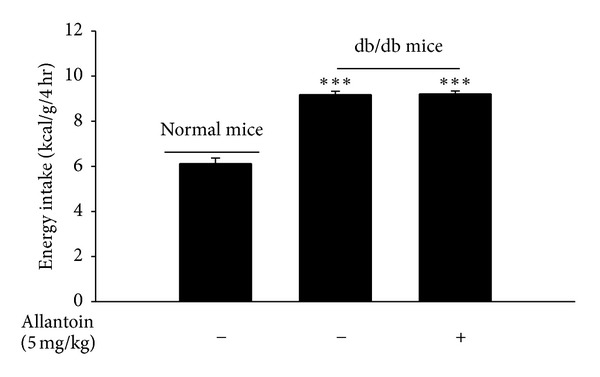
Loss of allantoin action in db/db mice. The db/db mice were treated with allantoin (5 mg/kg, i.p. three times per day) for 8 weeks. Then, the energy intake was measured and compared with another group that received injection of the same volume of vehicle. The values are expressed as the mean ± SEM from each group of eight animals ***P < 0.001 compared with the normal group.

As shown in Figure 5, energy intake was markedly increased in db/db mice. However, the injection of allantoin (5 mg/kg, i.p.) into db/db mice failed to produce changes in energy intake compared with vehicle-treated db/db mice. This indicates that the inhibitory effect of allantoin on energy intake disappeared in db/db mice.

### 3.6. The Improvement of Hyperleptinemia by Allantoin in HFD-Fed Mice

The administration of allantoin (5 mg/kg, i.p., three times/daily) for 8 weeks significantly decreased the plasma leptin level in HFD-fed mice (Table 2). This decrease was attenuated by pretreatment with efaroxan (1.5 mg/kg i.p.) 30 min before the administration of allantoin.

## 4. Discussion

In the present study, we found that allantoin caused a marked decrease in body weight and improved eWAT accumulation and energy intake in HFD-fed mice. This antiobesity action of allantoin was reversed by I_1_R blockade. These results indicate that allantoin may ameliorate HFD-induced obesity via the activation of I_1_R. 

Adipose tissue content is closely associated with obesity, and eWAT is widely used as an indicator in the investigation of obesity [18, 19]. In the present study, allantoin effectively decreased eWAT cell size (Figure 2) and weight (Table 1) via the activation of I_1_R. This action of allantoin on eWAT is effective at a dose similar to the dose required to improve hepatic steatosis in HFD-fed mice [16]. Allantoin can thus be considered to affect lipid homeostasis via the activation of I_1_R [16].

In addition to the role of adipose tissue, energy intake is also important in obesity. Some studies have indicated that imidazoline receptors exert beneficial effects on energy regulation [20, 21]. There are 3 types of imidazoline receptors, named as I_1_R, I_2_R and I_3_R [22, 23]. Previous studies have demonstrated that the activation of I_1_R may improve hypertension via sympathoinhibition [24]; the activation of I_2_R may improve insulin resistance via the AMP kinase pathway to enhance glucose uptake in type-2 diabetic animal models [25–27]; and the stimulation of I_3_R may stimulate insulin secretion from pancreatic *β* cells [28]. Although I_2_R has been reported to regulate monoamine oxidase (MAO) in the brain [29], I_3_R has not been found in the brain and is expressed mainly in the pancreas [28]. Thus, I_2_R and I_3_R seem unlikely to be involved in cerebral energy regulation. I_1_R is located in mainly the rostroventrolateral medulla of the brain stem, and it has also been identified in the hypothalamus [30, 31]. The activation of I_1_R has been reported to improve hypertension and hyperlipidemia [16]. Additionally, I_1_R activation was shown to attenuate hyperphagia in STZ-induced diabetic mice by lowering the hypothalamic NPY level [17], similar to the mechanism observed in Zucker rats [32]. Previous studies demonstrated that appetite was reduced by an increase in blood pressure [33, 34]. However, allantoin decreases appetite at a dose that is sufficient to lower blood pressure [16]. Thus, the change in blood pressure does not seem to be related to the change in feeding behavior caused by allantoin. In the present study, HFD-fed mice showed greater energy intake than did normal chow-fed mice (Figure 3). Allantoin attenuated this increased energy intake via the activation of I_1_R in HFD-fed mice, and this activity was reversed by I_1_R blockade with efaroxan [17]. The same dose of efaroxan alone didnot alter the appetite response in mice, indicating that alpha-2 adrenergic receptors, which may modify the feeding behaviors in animal [35, 36], are not involved in its action, and this result is consistent with previously reported data [17]. Clinically, imidazoline receptor agonist(s) are preferred to alpha-2 adrenergic receptor agonist(s) because they do not cause sedation as a side effect [37]. Additionally, imidazoline receptor agonist(s) showed no significant effects on general behavior in animals [38]. Moreover, the hypothalamic NPY level was also reduced by allantoin via I_1_R activation in HFD-fed mice (Figure 4). Thus, the effectiveness of allantoin in the regulation of energy intake is further characterized in this study. 

The adipocyte-derived hormone leptin has established roles in the regulation of energy intake, energy expenditure, and whole-body energy metabolism [39]. HFD-fed mice exhibited marked hyperleptinemia, consistent with a previous report demonstrating their inability to adequately respond to elevated leptin levels [40]. The present study showed that allantoin also decreased plasma leptin levels in HFD-fed mice via I_1_R activation (Table 2). Additionally, HFD-fed mice showed higher NPY levels, which may be associated with leptin signaling dysfunction [41–43]. To determine whether the action of allantoin on the inhibition of energy intake is mediated through endogenous leptin, we studied the energy intake in the db/db mouse, which is deficient for the leptin receptor [44]. As shown in Figure 5, allantoin failed to decrease the energy intake in db/db mice, due to the absence of leptin receptor in this strain. It has been reported that leptin can inhibit NPY secretion in the hypothalamus [45]. Thus, it is reasonable to speculate that the activation of I_1_R by allantoin may mediate leptin to inhibit hypothalamic NPY for reduction of hyperphagia to result in the decrease of obesity. However, the regulation of hypothalamic NPY level is quite complicated, including roles for proopiomelanocortin (POMC) neurons, the alpha-melanocyte-stimulating hormone (*α*-MSH), and melanocortin-3 (MC3) receptors [46, 47]. Thus, the detailed mechanism of action of allantoin in HFD-induced obesity requires further investigation in the future.

## 5. Conclusions

We found that allantoin has antiobesity effects in HFD-fed mice and that these effects are mediated by the activation of I_1_R, which results in lower energy intake and reduced eWAT accumulation. Thus, allantoin can be used as an alternative agent for the improvement of obesity in the future.

## Figures and Tables

**Table 1:** The inhibitory effects of allantoin on adipose tissue (eWAT) weight and ratio in HFD-fed mice. Efaroxan (1.5 mg/kg) was injected 30 min prior to the injection of allantoin (5 mg/kg).

	eWAT (g)	eWAT ratio (%)
Normal mice	0.85 ± 0.02	2.70 ± 0.07
HFD-fed mice		
+vehicle	2.04 ± 0.03***	4.00 ± 0.06***
+allantoin	0.89 ± 0.03	2.52 ± 0.06
+efaroxan and allantoin	2.02 ± 0.02***	3.94 ± 0.06***

The values represent the mean ± SEM of eight animals. The eWAT ratio was calculated by dividing the eWAT weight with body weight for each animal and was expressed as a percentage. ***P < 0.001 compared with the normal mice.

**Table 2:** The inhibitory effect of allantoin on hyperleptinemia in HFD-fed mice treated with allantoin for 8 weeks. HFD-fed mice received continuous administration of allantoin (5 mg/kg, i.p. three times per day), while another group was pretreated with efaroxan (1.5 mg/kg, i.p.) 30 min prior to the administration of allantoin.

	Leptin (ng/mL)
Normal mice	18.84 ± 1.36^###^
HFD-fed mice	
+vehicle	59.83 ± 1.05***
+allantoin	17.21 ± 0.82^###^
+efaroxan and allantoin	61.86 ± 2.25***

Values were obtained from each group of eight animals and expressed as the mean ± SEM. ***P < 0.001 compared with the normal chow-fed mice group; ^###^ *P* < 0.001 compared with the vehicle-treated HFD-fed mice group.

## Abbreviations

eWAT: Epididymal white adipose tissue
HFD: High fat diet
I_1_R: Imidazoline I_1_-receptors
IP: Intraperitoneal
NPY: Neuropeptide Y.
